# Limb-shaking transient ischemic attacks: case report and review of literature

**DOI:** 10.1186/1471-2377-6-5

**Published:** 2006-01-26

**Authors:** Saad Ali, Muhib Alam Khan, Bhojo Khealani

**Affiliations:** 1 Section of Neurology, Department of Medicine, Aga Khan University Hospital Karachi, Pakistan

## Abstract

**Background:**

Limb shaking Transient Ischemic Attack is a rare manifestation of carotid-occlusive disease. The symptoms usually point towards a seizure like activity and misdiagnosed as focal seizures. On careful history the rhythmic seizure like activity reveals no Jacksonian march mainly precipitated by maneuvers which lead to carotid compression. We here present a case of an elderly gentleman who was initially worked up as suffering from epileptic discharge and then later on found to have carotid occlusion.

**Case presentation:**

Elderly gentleman presented with symptoms of rhythmic jerky movements of the left arm and both the lower limbs. Clinical suspicion of focal epilepsy was made and EEG, MRI-Brain with MRA were done. EEG and MRI-Brain revealed normal findings but the MRA revealed complete occlusion of right internal carotid artery. On a follow-up visit jerky movements of the left arm were precipitated by hyperextension and a tremor of 3–4 Hz was revealed. Based on this the diagnosis of low flow TIA was made the patient was treated conservatively with adjustment of his anti-hypertensive and anti-platelet medications.

**Conclusion:**

Diagnosis of limb-shaking TIA is important and should be differentiated from other disorders presenting as tremors. Timely diagnosis is important as these patients are shown to benefit from reperfusion procedures either surgical or radiological reducing their risk of stroke.

## Background

Transient ischemic attacks typically present with neurological deficits such as loss of muscle power, reduced sensation, or loss of vision. Symptoms such as involuntary movements are not generally regarded to be a feature of cerebral ischemic episodes. Often confused with focal motor seizures, limb shaking TIAs are a rare form of TIAs that present as such and therefore pose a diagnostic challenge [1-6]. This distinction, however, is crucial as this form of TIA is often an indicator of severe carotid occlusive disease and patients are at high risk of stroke [5-8]. Moreover, such attacks may be relieved by surgical revascularization procedures in select cases [2,3,9] In the following, we present a case of limb shaking TIA and review the clinical manifestations, diagnosis, and management of this condition.

## Case report

A 65-year-old gentleman presented to us complaining of episodes of tremor of the lower limbs. These episodes would occur at 3–4 Hz, lasting for 1 to 2 minutes each in duration, and occur about once per month for the past 5 months, precipitated by standing up and extending the neck. At the time of the episodes, the patient also felt numbness in his left arm leading to decreased strength and a tremor of 3–4 Hz. Consciousness was never impaired and there was no other symptom. The patient had a past history of diabetes and hypertension, and was on anti-hypertensive and oral hypoglycemic medications. On examination the patient was found to have no neurological deficit. However, upon standing and extension of the neck, the patient became dizzy and felt numbness in his left arm. In addition, a swinging tremor of 3–4 Hz was noted in the same arm. These symptoms resolved on sitting.

Further investigations were needed. Interictal electroencephalography (EEG) and Video-EEG were normal, while EMG and nerve conduction studies revealed findings suggestive of mild peripheral sensory motor polyneuropathy. MRI of the brain was found to be normal; however, MRA of the anterior circulation revealed lack of flow signals in the right internal carotid artery consistent with complete occlusion. There was good compensatory filling of right anterior and middle cerebral arteries via cross flow from left internal carotid artery. Consistent with the above findings, Doppler ultrasound of the neck showed complete occlusion of the right internal carotid artery, with normal right common carotid, external carotid, and vertebral arteries. The diagnosis of low flow TIA was made.

The patient refused to undergo cerebral angiography for possible reperfusion intervention and was treated conservatively with antihypertensive and anti-platelet medications. Anti-lipidemics were also added and the patient hasn't complained of any symptoms for the past 6 months.

## Discussion

Since Miller Fisher's first description of temporary limb-shaking syndrome (LSS) associated with carotid stenosis in 1962, this diagnosis has been reported regularly [10] and to date there are about 45 such cases reported in literature [11]. The clinical features of LSS comprise a group of rhythmic or arrhythmic involuntary hyperkinesias affecting the hand, arm, leg, hand-arm, or hand-arm-leg unilaterally. When involving the hand-arm-leg, there is no Bravais-Jacksonian march and the face muscles are always spared; upper limbs are more evidently affected. Occasionally, jerking may be seen in both arms, asymmetrically, however. Most often, limb shaking is regular in frequency [2]. Patients describe movements as "swinging", "jerking", "twitching", "shaking", "trembling", inability to control the arm" and "lack of coordination". When observed by an examiner, LSS may assume either a choreic or a coarse tremor-like appearance. The shaking spells occur with a variable frequency; from single episodes to several times a day. Associated symptoms may include ataxia, myoclonic jerks, dystonic limb posturing, and parkinsonism, the latter manifested as micrographia, paroxysmal tremor and rigidity [11].

An almost invariable clue clinically is that symptoms arise after maneuvers that theoretically provoke cerebral blood hypoperfusion, such as arising from a bed or a chair, as well as hyperextending the neck. One patient had a repeated tendency to drop his key when leaving his car along with an associated tremor-like sensation and clumsiness of his left arm [12]. There is a short latency between standing and symptoms starting, usually of a few seconds. Conversely, limb shaking, lasting from a few seconds to minutes, ceases when the patient sits or lies down. These characteristics were present in our patient's historical account and we were able to elicit them in the clinic as well. Though not seen with our patient, other associated neurological features suggestive of the vascular nature of LSS are transient dysphasia and/or dysarthria, numbness of the shaking extremity, and ipsilateral hemiparesis.

According to Galvez-Jimenez et al., limb-shaking syndrome (LSS) is considered to be one of the many secondary or symptomatic dyskinesias of vascular etiology [13].

Like other vascular paroxysmal dyskinesias, it can be explained by the "hypoperfusion theory," in which carotid stenosis and orthostatism lead to decrease cerebral blood flow in critical watershed territories. In an assessment of 51 patients with infarcts in watershed cerebral territories, 12 percent were found to experience focal limb shaking by Bogousslavsky and Regli [14] In his physiological studies, Tatemichi et al used xenon-133 to detect regional decreases of cerebral blood flow and found significant hypoperfusion of the right dorsofrontal and upper rolandic regions contralateral to the shaking limb in a 63 year-old patient [4]. Baumgartner et al. used transcranial Doppler and showed reduced vasomotor reactivity to hypercapnia in all hemispheres opposite the involuntary limb movements in 5 patients. Later, in a another study, PET scan imaging revealed acetozolamide-induced hypoperfusion of corresponding cerebral territories in a patient with LSS, further suggesting that hemodynamic failure as the cause of transient ischemic attacks with limb shaking [5].

Electroencephalographic studies have failed to show epileptiform activity associated with limb-shaking syndrome, although some patients have contralateral slow activity [14]. Induction of repetitive involuntary movements in nine electroencephalographically monitored patients rendered abnormal findings in two; one had diffuse delta slowing and the other temporal delta slowing [2].

The most striking feature of LSS is severe internal carotid artery stenosis, necessitating the examination of carotid bruits in the elderly with orthostatic and/or episodic movement disorder. All 12 patients described by Yanangihara et al had occluded or critically compromised contralateral carotid artery [2]. Our patient was found to have complete occlusion of the internal carotid artery on the side opposite of the involuntary movements. Nonetheless, normal carotid angiographies with small vessel disease and discrete thalamic and midbrain infarction have also been reported in literature as a cause of LSS [13].

Management of low flow TIAs focuses on maintaining or improving cerebral blood flow by careful control of blood pressure and surgical revascularization. This divides the approach in surgical and medical modalities. In several cases an improvement of symptoms has been reported after raising blood pressure [6,10]. In the presence of concomitant cardiac and renal disease, this may be harmful. In such cases, more aggressive treatment of hypertension is possible after surgical revascularization, which is also effective in abolishing the attacks [1-5,7]. In patients with internal carotid stenosis, endarterectomy is the procedure of choice to abolish symptoms and reduce stroke risk [3,4]. In patients with complete occlusion, extra cranial-intracranial bypass surgery usually stops the attacks, though not reducing the stroke risk [3,9]. This is an old surgical technique which was abandoned after no outcome benefit was shown in a EC-IC bypass study [15]. However, the emergence of cerebral blood Flow monitoring techniques have brought EC-IC bypass in the front [16]. Currently, the potential use of superficial temporal artery to middle cerebral artery (STA-MCA) bypass for carotid occlusion is being re-evaluated by the Carotid Occlusion Surgery Study [17] and the Japanese EC-IC Bypass Trial (JET) [18]. These two studies will determine the fate of EC-IC bypass for carotid occlusion. Endovascular procedure for carotid chronic carotid occlusion has not been reported. However acute carotid occlusions have been stented following angioplasty showing favorable results but further studies are required for evaluation [19,20].

In summary, limb shaking is a rather uncommon form of transient ischemic attack that should be recognized and differentiated from conditions like focal motor seizures. We add to literature another case of this rare condition. Recognition will almost invariably point to carotid artery occlusion and timely treatment may not only abolish the attacks in patients but also reduce their risk of stroke.

## Competing interests

The author(s) declare that they have no competing interests.

## Authors' contributions

SA carried out the literature search of limb shaking TIA cases and collected all the data so far published. He then worked with the other two in writing down the paper.

MAK was involved in the initial diagnosis of the limb-shaking TIA and management of the patient. Later on worked with the two authors in manuscript preparation. He is the corresponding author as well.

BK is the treating neurologist of the patient and was involved in the initial diagnosis of the limb-shaking TIA and management of the patient. Reviewed the manuscript and helped in completing the final draft.

## Pre-publication history

The pre-publication history for this paper can be accessed here:


